# Co-designing the INHSU Prisons Hepatitis C Advocacy Toolkit using the Advocacy Strategy Framework^☆^

**DOI:** 10.1016/j.drugpo.2024.104628

**Published:** 2024-10-30

**Authors:** Shelley J Walker, Olivia Dawson, Yumi Sheehan, Lok B Shrestha, Andrew R Lloyd, Julia Sheehan, Nonso B Maduka, Joaquin Cabezas, Nadine Kronfli, Matthew J Akiyama

**Affiliations:** a National Drug Research Institute, Curtin University, Perth, Australia; b Burnet Institute, Melbourne, Australia; c Public Health and Preventative Medicine, Monash University, Melbourne, Australia; d The International Network on Health and Hepatitis in Substance Users (INHSU); e The Kirby Institute, University of New South Wales (UNSW), Sydney, Australia; f School of Biomedical Sciences, University of New South Wales (UNSW), Sydney, Australia; g The hepatitis C Trust, London, England, United Kingdom; h Bensther Development Foundation, Enugu, Nigeria; i Gastroenterology and Hepatology Department, University Hospital Marques de Valdecilla, Santander, Spain; j Clinical and Translational Research in Digestive Diseases, Valdecilla Research Institute (IDIVAL) Santander, Spain; k Department of Medicine, Division of Infectious Diseases and Chronic Viral Illness Service, McGill University Health Centre, Montréal, Québec, Canada; l Centre for Outcomes Research and Evaluation, Research Institute of the McGill University Health Centre, Montréal, Québec, Canada; m Department of Medicine, Albert Einstein College of Medicine / Montefiore Medical Centre, New York, USA

**Keywords:** Advocacy strategy, Hepatitis C virus, Hepatitis C elimination, Prisons, People who inject drugs, Advocacy

## Abstract

**Background:**

The World Health Organization (WHO) has established targets to eliminate the hepatitis C virus (HCV) by 2030. Prisons are a key focus of elimination efforts, however, access to HCV services in prisons remains low globally. With the aim of increasing advocacy efforts to help address this gap, the International Network on Health and Hepatitis in Substance Users (INHSU) Prisons, developed a Prisons Hepatitis C Advocacy Toolkit.

**Methods:**

Toolkit development involved a co-design process to ensure advocacy resources met end-user needs. A scoping study was conducted, involving a web-based survey and in-depth interviews, to understand advocacy resource needs of key stakeholders from countries of different socio-economic strata. Data were analysed, and suggested advocacy resources were mapped onto the Advocacy Strategy Framework with the audiences resources are targetting and the changes they aim to influence. Advocacy resources were co-developed and validated by interview participants before incorporation into the web-based platform.

**Results:**

Survey responses (*n* = 181) and interview data (*n* = 25) highlighted several barriers to enhancing HCV services in prisons globally, and an understanding that advocacy efforts are needed to bring about this change. Advocacy resources were suggested for influencing three key audiences: policymakers/funders, implementers, and community. Thereafter, a suite of 20 *de novo* tools were co-developed with key stakeholders including case studies of evidence-based models of HCV care, policy briefs, HCV infographics, and fact sheets about how to leverage funding and build advocacy campaigns. Findings underscore the importance of capitalising on the knowledge and expertise of potential end-users, to ensure Toolkit resources are context-specific and match their needs.

**Conclusion:**

The Toolkit holds promise for progressing the WHO elimination goals by increasing advocacy efforts for enhanced prison HCV services globally. The co-design of Toolkit resources with potential end-users has increased its potential accessibility, acceptability, and inclusivity for a globally diverse audience.

## Background

In 2016, the World Health Organization (WHO) established targets to eliminate the hepatitis C virus (HCV) as a public health threat by 2030 (WHO, 2016). Although significant progress towards this goal has been achieved, of the estimated 50 million people living with HCV globally in 2022, only 36 % (18 million) had been diagnosed, and 20 % (12.5 million) had received curative treatment, which is far below the global targets for eliminating HCV by 2030 (WHO, 2024).

The sharing of injecting equipment among people who inject drugs accounts for the largest number of new HCV infections in the world, with unsafe injecting drug use practices estimated to contribute 43.6 % (33.9 % – 52.5 %) of new HCV infections globally (WHO, 2024). Due to the criminalisation of drug use in most parts of the world, it is estminated that up to two thirds of people who inject drugs have ever been incarcerated (Degenhardt et al., 2023; Stone et al., 2018) and that 1.5 million people in prison are living with chronic HCV infection (Dolan et al., 2016). Furthermore, prisons are high-risk environments for HCV transmission because access to harm reduction measures such as needle and syringe programs (NSPs) and opioid agonist therapy (OAT) remains low (HRI, 2023). Given the magnitude of the population of people either living with chronic HCV or at risk for HCV in prisons, carceral settings are widely recognised as crucial for enhancing access to HCV testing and treatment (Akiyama et al., 2021, 2022; Pawlotsky et al., 2020; Stone et al., 2018; et al., 2022).

While it is estimated that two thirds of countries globally have national HCV testing and treatment plans in place, in 2019 only one third (35 %, *n* = 28) ) of 81 national plans included carceral settings as a focus—including 12 in high-income countries (HICs)—underscoring a critical lack of attention to people in prison (Akiyama et al., 2022; WHO, 2019). Moreover, while many HICs offer universal and subsidised access to HCV testing and treatment in the community, access to HCV healthcare (including systematic HCV screening, free DAA treatment, prison-based NSPs, community HCV transition programs) in carceral settings remains suboptimal (Kronfli et al., 2021; Ocal et al., 2019; Stöver et al., 2019). Several factors contribute to these HCV service gaps, including: lack of political will and commitment, limited HCV prevalence data in prison populations, the prioritisation of prison security over healthcare needs, scarcity of funding and resources (i.e., for diagnostic equipment and direct-acting antivirals), overcrowded prisons, stigmatisation of people who use drugs and people living with HCV, lack of knowledge about HCV, prison infrastructure logistical challenges, and staff shortages or competing priorities (Akiyama et al., 2021; Nakitanda et al., 2020; Papaluca et al., 2019).

Advocacy efforts are needed to increase access to HCV testing and treatment in prisons if HCV elimination targets set by the WHO are to be reached (Akiyama et al., 2021; Rockstroh et al., 2023; Walker et al., 2024; Winter et al., 2022). Toolkits that focus on increasing advocacy efforts for the elimination of HCV globally are widely available (Caring Ambassadors Program, 2022; Centre for Alcohol & Other Drug Training & Workforce Development, 2023; HepVu, 2023; National Alliance of State & Territorial AIDS Directors, 2011; World Hepatitis Alliance, 2018), however, none focus specifically on prisons.

## Advocacy for enhanced HCV services in prisons globally

Advocacy is internationally recognised as a core public health strategy for redressing health inequities (Avery & Bashir, 2003; Cohen & Marshall, 2017; Loue, 2006). At the heart of advocacy is the assumption that change can happen through building public awareness, presenting evidence for the reasons why change should happen, giving voice to those who are affected, and engaging people who have the power to make those changes (Avery & Bashir, 2003). Although the idea that advocacy is important for addressing health inequities is supported theoretically, its role in practice has not been fully realised due to multiple constraints (Cohen & Marshall, 2017). Identified barriers include poor education and training of health professionals and public health practitioners about how to frame advocacy issues, insufficient data or information to support advocacy efforts, and an overall lack of interdisciplinary collaboration and organisational support to pursue advocacy activities (Cohen & Marshall, 2017; Laari & Duma, 2023).

The International Network on Health and Hepatitis in Substance Users (INHSU) Prisons, developed a Prisons Hepatitis C Advocacy Toolkit (herein referred to as the ‘Toolkit’) to address these barriers. The Toolkit consists of a web-based repository of evidence-based, user-informed resources to support and motivate stakeholders to advocate for improved HCV services in prisons globally. In this article, we describe the co-design process used to inform the development of advocacy resources for inclusion in the Toolkit and introduce the Prisons HCV Advocacy Toolkit.

## Research methods

Research methods are described below, via the three steps in our co-design process.

Ethics approval for the study was received from the Institutional Review Board of the Office of Human Research Affairs at the Albert Einstein College of Medicine (IRB #: 2023–14716) in March 2023.

### The co-design process

Increasing the participation of key stakeholders in the process of designing solutions to public health problems can strengthen the innovation, implementation, and overall success of population health initiatives (Ramaswamy & Ozcan, 2014; Slattery et al., 2020; Vargas et al., 2022). With the aim of achieving these outcomes for the Toolkit, we involved potential end-users in its co-design, a process that involved individual and repeated interactions with a diverse group of key stakeholders. Three key steps were involved: 1) a mixed methods scoping study to understand the global advocacy resource needs of diverse key stakeholders; 2) identifying target audiences and mapping suggested Toolkit resources using the Advocacy Strategy Framework (Coffman & Beer, 2015); and 3) co-developing and validating advocacy resources for inclusion in the Toolkit. Table 1 provides a summary of the co-design steps.

#### Stage 1. Scoping study to determine advocacy needs

The first stage of the co-design process involved a mixed methods scoping study (Walker et al., 2024), which aimed to understand what prison-based HCV services are available globally; barriers for enhancing prison-based HCV services globally; advocacy efforts needed to address barriers; and suggested Toolkit resources to facilitate advocacy efforts. See Walker et al., 2024 for more detail.

##### Quantitative survey

A short web-based survey (Survey Monkey Inc., 2023) was developed and distributed to targeted key stakeholders who are involved in the development, delivery, and/or oversight of prison-based health services. Two rounds of the survey were distributed via INHSU Prisons e-mail membership and e-newsletter lists (*n* = 3763) between March and April 2023. Participation was voluntary and involved implied consent via survey completion. The names of participants were entered into a draw for complimentary registration to the INHSU 2023 Conference. Data were extracted into Microsoft Excel and analysed using R Studio. The association between independent categorical variables was calculated using Chi-square or Fisher exact tests. P values of less than 0.05 were considered statistically significant.

##### Qualitative in-depth interviews

Qualitative in-depth interviews were conducted with key stakeholders from ten countries between June and September 2023 to gain more nuanced global understandings of domains of investigation in the survey. Participant recruitment was informed by survey responses, which involved identifying countries with survey responses from at least three individuals and, from these, selecting ten geographically dispersed countries of different socio-economic strata.^2^ We purposively selected four low-income countries (LICs); three middle-income countries (MICs); and three HICs. Invitations to participate in an interview were emailed to at least three purposively selected survey respondents in different professional roles from each country. A semi-structured interview schedule (Supplement S1) guided conversations that were tailored to individual needs using survey data as prompts. Interviews lasted an average of 47 min. Participants were offered an honorarium of $USD50 for their time and expertise. Interviews were audio-recorded and transcribed verbatim and data were organised using NVivo data software (QSR International, 2020). We thematically analysed data using an iterative approach, which involved developing key deductive codes based on research aims, followed by inductively refining, collapsing, and expanding codes as new and emergent themes were identified (Clarke & Braun, 2017). Coded data were exported into Word documents and extracts were synthesized and refined to produce final themes and sub-themes.

#### Stage 2. Identifying target audiences and mapping resources using the Advocacy Strategy Framework

It is widely acknowledged that for advocacy efforts to be effective, they should be grounded in research evidence and an understanding of the actions needed to achieve the desired change (Klugman, 2011). Conceptual frameworks can help identify desired outcomes, assist in determining who needs influencing and how, ensure that a clear rationale exists regarding why activities are expected to lead to certain outcomes, and ensure outcomes can be measured (Glass, 2017). Several frameworks have been created to assist the development and evaluation of public health advocacy initiatives (Glass, 2017; Klugman, 2011). Many have been criticised, however, for obscuring rather than illuminating the critical components of an advocacy strategy, for example being too linear or complex to navigate; too removed from context; or too restricted in thinking about how strategies need to adapt over time (Gen & Wright, 2013; Gill & Freedman, 2014).

For the purposes of supporting our co-design process, we chose Coffman and Beer’s (2015) Advocacy Strategy Framework (Supplement S2), which was designed for thinking through and articulating how change is expected to evolve over time, and the role individuals and organisations play in creating that change. The Framework is represented via a grid and organised around two key dimensions considered critical for success in advocacy: ‘audiences’ and ‘changes’ (Glass, 2017). The horizontal axis represents the three ‘audiences’ the advocacy strategy is targeting and attempting to influence, and the vertical axis represents the three ‘changes’ needed to progress towards the desired ‘goal’. Drawing on in-depth interview data from Stage 1, the Framework was adapted and used to help identify the target audiences of the Toolkit resources and to map the position of the resources on the grid, both with respect to audiences and the continuum of change.

#### Stage 3. Co-developing and validating advocacy resources

The final phase of the project involved co-developing and validating resources with scoping study participants, to ensure they matched the needs of potential end-users (including the three audiences targeted) and had broad applicability across different socio-economic strata (including for LICs, MICs and HICs). This process was led by the Toolkit and INHSU staff with skills in online learning development/design and media communications and marketing. Web design development was outsourced.

Informed by findings from Stage 1, we drew on in-depth interview data to create ten tailored lists of 7–12 resources (one for each country). Lists of resources (including a description of each resource) were emailed to participants (*n* = 25), and a first round of feedback was sought via SurveyMonkey and email correspondence, to determine if the proposed resources were appropriate (including the types of resources and their content) for the purposes of advocacy. Three questions were included in the survey: 1) ‘I think the tools suggested (i.e. video/fact sheet, etc.) will meet the needs of advocates in my country who are working to scale up HCV testing and treatment services in prisons’; 2) ‘I think the content of the tools suggested will meet the needs of advocates in my country who are working to scale up HCV testing and treatment services in prisons’; and 3) ‘Overall, I think the advocacy tools suggested are appropriate for my country setting’, with responses reported via a sliding scale (strongly agree / agree / neither agree nor disagree / disagree / strongly disagree / free text response).

A suite of draft resources was developed based on participant feedback, and participants were invited to provide a second round of feedback via SurveyMonkey and/or a comprehensive offline review (including financial reimbursement of up to two hours), regarding resource content (i.e., language, messaging) and user-friendliness (i.e., design, format). Following the incorporation of this feedback, a third/final round of participant input was invited via email, and resources were adapted where needed and finalised for approval by the project team for inclusion in the web-based platform of the Toolkit.

## Results

Scoping study results are presented below, followed by our use of the Framework to identify target audiences and map advocacy resources, and the process used to co-develop and validate Toolkit resources. Finally, we introduce the Prisons HCV Advocacy Toolkit.

### Stage 1. Scoping study

#### Quantitative results

Survey responses were received from individuals in 41 countries (38 % of 108 countries of survey recipients), including 18 LICs, 6 MICs, and 17 HICs, with an overall response rate of 4.8 % (181/3763). Most responses (61 %, *n* = 110) were from HICs, 30 % (*n* = 55) were from LICs, and 9 % (*n* = 16) were from MICs. Almost half of respondents (47 %) identified as health program implementers including nurses, general practitioners, hepatologists, and gastroenterologists; over one-third (37 %) held professional roles such as advocates, academics, harm reduction workers, peer workers, or community health service providers; 10 % identified as policymakers; and 6 % provided funding for health and/or prison sector programs. Approximately half (52 %, *n* = 94) of survey respondents reported working in a custodial setting (including community-based organisations operating in prisons).

According to survey responses, the availability of prison-based HCV services offered in prisons differed considerably across countries of different socio-economic strata. For example, all prison-based HCV services were more likely to be available in HICs than LICs, including venipuncture-based HCV antibody testing (88 % HICs vs 33 % LICs), genotyping (78 % HICs vs. 24 % LICs), direct-acting antivirals (88 % HICs vs. 38 % LICs), and post-treatment follow-up (76 % HICs vs. 27 % LICs) (Walker et al., 2024).

Suggested advocacy resources are shown in Table 2. These included best practice case studies in video format, fact sheets, and advocacy strategy guidelines; the latter were significantly more likely to be reported in LICs compared to HICs and MICs (p-value <0.001). Social media templates were twice as likely to be suggested by participants in LICs compared to HICs and MICs (p-value 0.003).

#### Qualitative findings

Interview participants included nine policymakers; nine implementers (including medical doctors, infectious disease physicians and hepatologists, nurses, and prison staff); four “advocates” (including managers of health services and peer workers); and three researchers working in academic institutes. As highlighted above, interviewees were from four LICs (Indonesia, Kenya, Nigeria, Pakistan); three MICs (Moldova, South Africa, Thailand), and three HICs (Greece, United Kingdom, United States).

Qualitative findings are presented below via the key domains of the Advocacy Strategy Framework, including the three targeted audiences (policymakers/funders, implementers, and community) advocacy efforts aim to mobilise and influence, and the change needed (awareness, will, and action) to bring about the desired goal, i.e., enhanced prison-based HCV services globally. Additional qualitative findings are published elsewhere (Walker et al., 2024). See Table 3 for a list of suggested advocacy resources by target audience and changes needed.

#### Policymakers and funders

All interview participants identified policymakers/funders of HCV programs and services as one of the most important audiences for Toolkit resources, including: national or jurisdictional level decision-makers; key stakeholders in departments of health, corrections and justice health; and prison governors and senior level administrators.

#### “Awareness” of the importance of HCV programs in prisons

All participants recognised that implementing or enhancing HCV services in prisons required an awareness by policymakers/funders that HCV is a public health problem that should be prioritised.

Participants described the challenges of having to compete with other health priorities that governments and funders considered more important. In low- and middle-income countries (LMICs), where access to HCV services in prisons is limited, participants described needing basic information in the form of policy briefs to advocate to those in positions of power and influence.

If we’re talking about advocacy, first, we need a policy brief for the politician to understand the issues in one page, in simple words. Maybe a few graphs, a few numbers, and the message needs to be that if you spend the money here, you will save money there, in the long term. Yes, we are looking for a win-win situation when we are talking about advocacy. (P9, Academic, MIC)

While data about the prevalence and economic benefits of HCV testing and treating in the general community was said to be mostly available, for prisons this was not the case. Thus, participants across all countries spoke extensively about the need for HCV prevalence and cost effectiveness modelling data as a tool for convincing policymakers/funders about the need for HCV services in prisons.

Since 2016 when the DAAs became available we’ve had excellent data more broadly on, you know, the health and economic benefits of treating hep C, and we’ve got good data on the reduction in decompensated liver disease and hep C at the population level. But we haven’t got any of that data that specifically relates to the prison population, and I think, in terms of advocating to the higher levels of government for more spending in this area - because it’s certainly not an area in the health sector budget that receives the highest priority – we need that data. (P1, Implementer, HIC)

#### Political “will”

Many spoke with frustration of knowing that prison HCV prevalence data were one of the most important advocacy tools for convincing and gaining the support of governments, decision-makers and funders, but that gathering this data in their own country could only be achieved with funding to test people.

We need data for advocacy because the government, they will ask, “what is your evidence?” Because in a low-income country [...] their funding priorities are maternal health, children’s health [and] to get the chunk of the funding [...] the thing we need is the baseline data of viral hepatitis in prisons. We have data about injecting drug use in the prisons, but the government they want to see, not only how many are injecting drugs, but how many have hepatitis C, and we cannot get that data if we cannot test (P16, Advocate, MIC)

Lack of funding was considered a major barrier to implementing or scaling up HCV services in prisons in most countries, thus, advocacy resources that could help Toolkit users access funding were suggested, particularly for LMICs. For those in HICs where HCV services in prisons were already being delivered, tips on how to access additional funding was suggested for program expansion and sustainability.

I think there should be a section on how to find funding. Like, are there pots of money you could be accessing ... and where are they. And grant writing that can bring in money is not easy, and so I think there should be a section on what funds are available for your country. (P18, Advocate, HIC)

Although all participants understood the importance and benefits of advocacy efforts to enhance prison-based HCV services, in LMICs, some suggested resources were needed to help map and plan advocacy processes, including “who you should be talking to in government”, and “what you need them to know”:

Because when we talk about advocacy, there are different stakeholders, so stakeholders mapping should be a critical aspect of the toolkit - who should we reach out to would be very, very important. (P16, Advocate, LIC)

#### “Action” to implement HCV services in prisons

Advocacy resources that showcase successful HCV programs and initiatives implemented in other countries of similar socio-economic strata were described as an important advocacy tool to motivate policymakers and funding bodies to act.

We can push the relevant authorities and policymakers to say that, for example in [this country] this is what they have done, so they can see that it has been done before and maybe we can do it too. (P15, Implementer, MIC)

Several HIC participants described how the evidence they had gained via the implementation and evaluation of successful HCV prison programs, had been critical for gaining additional funding. In LMICs, however, a common reported barrier for increasing HCV programming in prisons was a lack of expertise or resources to monitor and evaluate pilot programs. To address this gap, participants suggested “how to” resources were needed for monitoring and evaluating HCV services in prisons.

We’re very familiar with how to collect data for the management of the AIDS program. We did that very well [because] we have one of the best national AIDS program data recording systems. However, our people who work in hepatitis C, they strived so hard to get a grant for a data management system for hepatitis C [because] we know the benefit of knowing the success of your implementation. We want to know, what is the proper or essential data, what data is needed, and how can we implement a tracking system for the work we do, that doesn’t increase burden on people in the field. (P17, Advocate, MIC)

#### Implementers

A wide variety of individuals involved in HCV program implementation in prisons were identified as key audiences for the Toolkit resources, including nurses, medical doctors, infectious disease specialists, volunteer carers, and prison officers.

#### HCV “awareness”

Across all countries, to varying degrees, participants shared concerns about a lack of knowledge and awareness about HCV among people implementing programs in prison (including transmission routes, diagnostic and treatment options, and the importance of testing and treating people in prison).

In LMICs, a common concern was a lack of formal or up-to-date training about BBVs for health care personnel. Basic information about HCV, such as “viral hepatitis transmission modes”, and “how to diagnose” HCV, via video format or factsheets, were suggested for addressing this barrier.

The knowledge gap is a big issue in our country [...] Even when you talk to the doctors who work in the prisons, they know very little about hepatitis [...] which is why we need education first, because not so many people who provide healthcare in our prisons are aware about the management and treatment for viral hepatitis. (P2, Policymaker, LIC)

Across countries of all socio-economic strata, participants described needing resources to address the stigmatising attitudes of health care personnel and prison/security guards. Suggested resources to help “change their mindset”, included downloadable factsheets and infographics to debunk myths and misconceptions.

Advocacy tools for the prisons and the security staff would be good, to help us to sort of change their mindset, get them thinking about health, you know, and why it is important that we test and treat people in prisons. (P1, Implementer, HIC)

Resources to educate health care practitioners about the importance of screening blood products and sterilising medical equipment were also suggested in LMICs, given their high rates of HCV transmission via the use of unsterile medical instruments and procedures in healthcare settings.

Awareness raising has to be done about the improperly screened blood [and] sterilized equipment because many people who even work in healthcare, they claim they know, but they do not know these things about hepatitis C [...] Awareness raising, it has to be a priority, because people need to know that there’s a difference between using soap and water, and why sterilization can prevent this disease. (P10, Policymaker, LIC)

#### “Will” to provide HCV services in prisons

To gain the enthusiasm of health care staff in prisons to test and treat people for HCV, participants across all countries suggested advocacy resources were needed “about interventions that had worked [and] success stories”.

A summary table of work being done in [prisons] in other countries would be good, because we always have to discover everything from the start, from scratch. For example, how is testing implemented in other places for people when they enter prison. What about opt in or out programs, and what are the advantages of these approaches ... especially successful examples. (P20, Implementer, HIC)

Several participants in LMICs described how being able to learn and “borrow ideas” via case studies of successful programs implemented in other countries of similar socio-economic and political status, would be useful for inclusion in the Toolkit.

If a study’s been done in [another country] and we have a similar social, economic and political environment, then maybe it’s something we can learn from for our country. Like what are the valuable studies globally and in different regions, so if there’s no studies in [our country] maybe we can borrow ideas from prisons in other places. (P14, Policymaker, MIC)

Participants from LMICs also described the importance of being able to network and connect with others delivering HCV programs in prisons, given most lacked funds to attend international conferences or forums. They viewed the Toolkit as an opportunity for helping overcome this barrier, by providing information about organisations implementing HCV services in prisons.

We didn’t just come up with this idea for the prison project out of the blue, like “let’s do a prison project”. We learned this from another organisation we met through a regional learning platform. And as we talked, we thought, “oh, they can do this, maybe we can also do this [on] a smaller scale”. So that’s how we got the idea of how to initiate the project and what to consider (P6, Advocate, LIC)

#### “Action” to provide HCV services in prisons

Many participants described how despite having the resources to test and treat people for HCV in prison, a lack of clinical skills, confidence, and expertise among health care providers were barriers that prevented the effective delivery of their services.

In LMICs, several participants said health care providers lacked up-to-date clinical guidelines, and that access to information about HCV cascades of care and best practice procedures for HCV *screening, diagnosing and prescribing, would be useful Toolkit resources.*

We have guidelines for communicable diseases, but they’re only distributed to the specialists, [and] I think there’s new updated guidelines that we do not have. Ours are from 2018 which is an issue for us [...] The guidelines should not only be about medication for the physician or lab information, there should also be information to educate the nurse about how to ask about risk factors [...] how to screen, and how to communicate with the patient. (P13, Implementer, MIC)

To advocate for “better care” for people in prison with HCV, resources aimed at enhancing non-judgmental and empathic communication skills and practices of healthcare professionals were suggested.

I know we’re often accusing doctors of not doing this or not doing that, but some of them will say, “none of us were actually trained on how to do it, you just expected us to be able to interact with all these different levels of different diseases, and different people”, and so just basic kind of guidance [...] in terms of language that would be more appropriate [and] empathetic. (P3, Policymaker, LIC)

Given other BBVs such as HIV and hepatitis B are highly prevalent among people in prisons in LMICs, and they share similar HCV prevention measures, several participants suggested resources pertaining to other BBVs should also be included in the Toolkit.

One of my recommendations for the Advocacy Toolkit is that hepatitis C should not be looked on in isolation. It should include hepatitis B, because they share transmission routes in LMICs and many of the prevention dimensions can be coupled, and hepatitis B is often more endemic. (P12, Academic, MIC)

#### Community

The third key audience participants identified as important for targeting advocacy resources was the community, including individuals working in HCV transitional care services, harm reduction organisations, and peer organisations working with people who inject drugs and people at risk of or living with HCV in prison and the community.

#### HCV “awareness”

Across countries of all socio-economic strata, poor knowledge about HCV including transmission routes, symptoms, and potential health consequences, was considered a key barrier that prevented people in prison choosing to get tested and treated. Therefore, people in prison at risk of or living with HCV were considered an important target audience for advocacy efforts.

Posters with facts about hepatitis, maybe for the walls so the inmates can learn about hepatitis [...] because not so many people in prison are aware - it could really help them to make decisions about getting tested. So, this is one of the things we would like to see in the Toolkit. (P2, Policymaker, LIC)

Across all countries, short videos, fact sheets, and downloadable infographics and images for posters or small booklets, were offered as suggestions to raise HCV awareness among people in prison, to help dispel myths and misconceptions, and to build an understanding of the importance of testing and treatment during incarceration.

If you have lots of infographics in the Toolkit, that they can use in posters or flyers, but also downloadable hep C posters and flyers – because essentially, we can’t advocate for more people to be tested and treated, if the people in prison don’t know why they should be tested and treated. (P18, Advocate, HIC)

Importantly, participants across all countries said poor literacy levels of people in prison meant resources should be visual and engaging and avoid written text.

One of the big issues we have in prisons is that lots of prisoners can’t read, so written information is not ideal, and so videos and films can really help with that issue – and I think even for those who can read, written material isn’t very engaging. (P1, Implementer, HIC)

#### “Will” to get HCV tested and treated

Peer programs to educate, support, and provide HCV care to people in prison at risk of or living with HCV were identified as a powerful mechanism for advocating to people in prison to get tested and treated, thus case studies of successful peer-led HCV programs in prison were suggested for the Toolkit.

It would be brilliant if part of the toolkit could be about peer-based models in prisons, so if an organisation has a little bit of money you could map out how to use the money for peer programs [...] Having people with lived experience involved in delivering programs - even if it’s just educating people in prison about hep C, is a relatively cost-effective way of raising awareness, encouraging people to get tested, changing the conversation and dispelling myths. (P18, Advocate, HIC)

Given the documented successes of peer-led programs, several participants suggested stories of people who had been tested and treated for HCV in prisons would be useful, for increasing the motivation and will of others to get tested and treated.

Real-life stories of individuals who have undergone treatment and improved their health I think would be useful as they can inspire other people in prison to get tested. (P24, Policymaker, MIC)

#### “Action” to motivate HCV testing and treatment

Most participants described challenges ensuring people treated in prison had access to follow-up care after release. In HICs where follow-up HCV services in the community were usually available, poor “communication between the two primary care systems - prison health and healthcare in the community” was reported as a barrier, and thus tips on how to address this were described as needed.

Many people are [released from] prison before they finish treatment – we have a lot of these cases - and it’s very challenging because they have a lot of problems to deal with when they’re released, and so health is often hardly their priority. But we are trying hard to find a way to connect them to services on the outside, so they get that care after they leave, so tips on what’s worked for others doing this work would be good. (P17, Advocate, MIC)

In some LMICs, another reported barrier to ensuring people complete their HCV treatment after release from prison, was a lack of available HCV programs in the community. Case studies of initiatives that had capitalised on services in the community for people with HIV were suggested to address this barrier.

We have many programs in the community for people with HIV, so to know how we can work with these services to test and treat for hepatitis C would be helpful. (P6, Advocate, LIC)

### Stage 2. Identifying target audiences and mapping resources using the Advocacy Strategy Framework

Drawing on qualitative data from Stage 1 and the Advocacy Strategy Framework, we established three target audiences for Toolkit resources (as highlighted above), including: 1) ‘policymakers/funders’ (e.g., governments, political leaders, senior level decision-makers); 2) ‘implementers’ (e.g., prison-based healthcare providers, doctors, nurses, prison security staff); and, 3) ‘community’ (e.g., peer organisations, harm reduction services, post-release programs for people at risk of or living with HCV). We retained Coffman and Beer’s (2015) stages of change, including: 1) ‘awareness’ (to create awareness that HCV is a problem that needs addressing for people in prisons); 2) ‘will’ (to raise the audiences’ willingness to take action to enhance HCV services in prisons); and 3) ‘action’ (to increase policy efforts that support and facilitate this change).

A list of suggested advocacy resources was compiled according to the audiences the resources were meant to target and the changes needed to enhance prison-based HCV services globally. Suggested resources are presented in Table 3, including their relevance for LICs, MICs, and/or HICs.

**Fig. 2.**
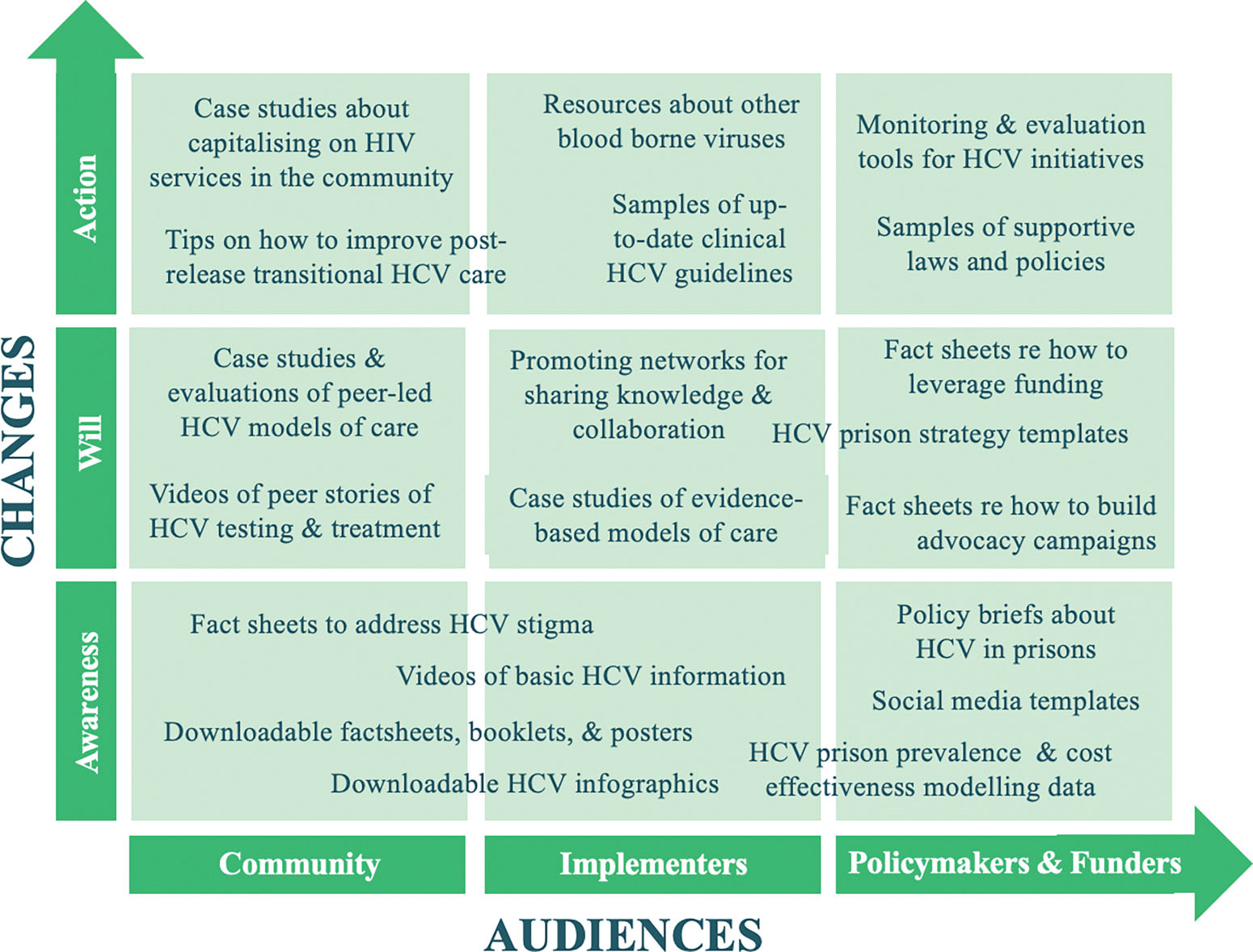
Suggested resource mapped onto the Advocacy Strategy Framework.

Suggested resources were mapped onto the Advocacy Strategy Framework according to the three Toolkit audiences, and where they fit on the continuum of change (Fig. 2).

### Stage 3: Co-developing and validating advocacy resources

**Fig. 3.**
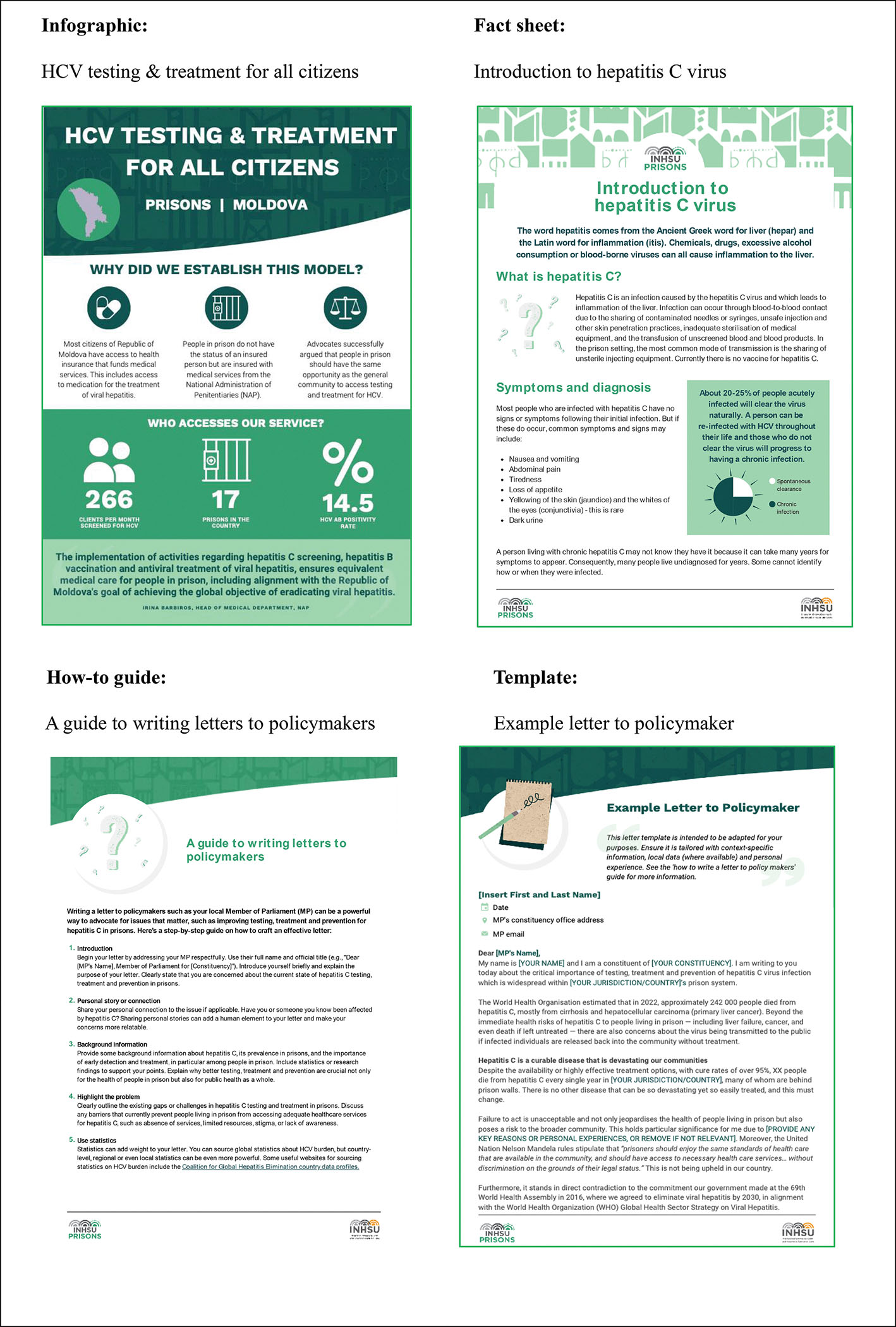
Sample Toolkit resources.

A total of 21 (84 %) participants reviewed and provided feedback on draft resources over the three rounds of review (LICs, *n* = 6; MICs, *n* = 8; HICs, *n* = 7); feedback from each round informed the next stage in the development process. Most participants found the lists of tailored resources generally suitable for their country context (round 1), with only minor suggestions made which were mostly related to the need for additional content (e.g., information requested about how to maintain people on HCV treatment after their release from prison). Most Toolkit resources were developed *de novo*, however, we also sourced some pre-existing resources (e.g., infographic templates, factsheets, and videos about how HCV affects the liver) and some web content inspiration was drawn from existing INHSU HCV infographic templates and online learning modules, and the Nohep Race to 2030 Advocacy Toolkit (World Hepatitis Alliance, 2018; International Network on Health and Hepatitis in Substance Users, 2024). In the second and third rounds of review, participants provided more constructive feedback, including suggestions related to resource content or the removal of unnecessary text. Content specific suggestions were about scope (e.g., acknowledging the lack of access to FibroScan diagnostic equipment in some country settings); use of terminology (e.g., ensuring acronyms such as Medication Assisted Treatment, Opioid Agonist Treatment or Opioid Substitute Treatment were used appropriately according to country context); requests for additional country specific information (e.g., information about how to address treatment initiation barriers for undocumented citizens in prisons); or the addition of topics felt to be missing (e.g., more examples of funding sources for prison-based HCV programs, or more examples of data collection tools for monitoring and evaluation purposes). In addition, several (*n* = 8) in-country key stakeholders directly participated in the content development of videos and infographics as they pertained to in-country case studies. A final suite of 20 resources were developed de novo (https://www.hcvprisonsadvocacy.org) including seven fact sheets (e.g., stigma and discrimination, HCV prevention, prison language guide, monitoring and evaluation); six how-to guides and templates (e.g., how to write a press release or letter to parliament, social media templates); five videos, including case studies in one LIC, one MIC, and three HICs; and two infographics of country-specific best practice models of care. Four sample advocacy resources are provided in Fig. 3.

## Discussion

Our study examined the perceived barriers to scaling-up HCV services in prisons globally, to inform the development of a targeted advocacy toolkit, for progressing HCV elimination efforts. Scoping study findings affirmed that many people at risk of or living with HCV in prisons globally do not have access to HCV testing and treatment, especially in LMICs (see also Walker et al., 2024). Data on suggested advocacy resources provided further evidence of the multiple barriers that exist for scaling up prison-based HCV testing and treatment services (Akiyama et al., 2021, 2022; Kronfli et al., 2019; 2021; Lafferty et al., 2018; 2021; Nakitanda et al., 2020; Papaluca et al., 2019). Our study affirmed, however, that significant support and enthusiasm exist for increasing HCV testing and treatment services in prisons globally, and that advocacy efforts are desired and needed to bring about this change.

It is not surprising that participants in LMICs were more likely than those from HICs, to request advocacy resources for gaining the support of policymakers and funders, as prison-based HCV services are less likely to be prioritised and adequately resourced in LMICs (Akiyama et al., 2021, 2022; Walker et al., 2024). These findings highlight, however, that much work is needed to garner their motivation and support, including at the very least, building their awareness that HCV poses a significant health burden on those who are affected, that people in prisons are more likely to be affected, and that there is a simple cost-effective solution to addressing this public health problem. Tailored Toolkit resources such as policy briefs, prison strategy templates, fact sheets about HCV and how to build advocacy campaigns, have the potential to begin this awareness raising process in LMICs (Akiyama et al., 2021, 2022). As LIC participant narratives highlight, such resources are also needed to leverage funding for pilot programs, ensure direct-acting antiviral treatments are, at the very least, available for those who have chronic HCV infection, and to develop monitoring and evaluation systems to measure the success of such initiatives.

Although our data reflect that many HICs are comprehensively providing HCV services in prisons, including a few that have reached elimination in individual prisons, they also highlight that resource barriers still exist before WHO elimination goals are met in HICs (Kronfli et al., 2021; Nakitanda et al., 2020; Papaluca et al., 2019). It is hoped that the inclusion in the Toolkit of prison HCV prevalence and cost analysis modelling data, which were described as needed in all countries including HICs, will help advocates leverage funding to address issues such as healthcare workforce shortages and a lack of access to diagnostic equipment such as point-of-care RNA testing (Kronfli et al., 2019; 2021; Lafferty et al., 2018; Papaluca et al., 2019).

The desire for resources that can tangibly support the building of HCV knowledge and clinical skills and expertise for implementers of health services in prisons in LMICs is a key finding. Samples of up-to-date HCV clinical guidelines and case studies of evidence-based models of HCV care, as suggested and co-designed by participants, have the potential to address these barriers, by helping build healthcare provider knowledge and confidence in delivering HCV services. Furthermore, the inclusion of awareness raising resources such as videos and downloadable posters and factsheets about HCV, have the potential to help dispel misconceptions about HCV and address stigmatising attitudes that continue to prevent HCV testing and treatment uptake in countries of all socio-economic strata. Case studies and evaluations of peer-led models, and videos of positive stories of testing and treatment experiences in prisons from peers, also have the potential to support these advocacy efforts.

Grounding our resource development in the Advocacy Strategy Framework helped us identify and specify key audiences (i.e., policymakers/funders, implementers, community stakeholders), consider the various stages on the continuum of change for achieving our aim of increased HCV services in prisons globally (i.e., awareness, will, action), (Coffman & Beer, 2015). The user-friendly, flexible, non-linear and multi-dimensional features of the Framework supported us to think about how these audiences and changes are positioned in relation to each other (Glass, 2017). The co-design process we used, which involved using both survey data and interview findings from diverse key stakeholders globally, helped to ensure advocacy resources align with stakeholder needs (Sánchez de la Guía et al., 2017). The process also allowed us to tailor resources to match the various political, cultural, and socio-economic country contexts, and the various carceral environments that exist within each country. Capitalising on key stakeholder knowledge and expertise provided opportunities to promote and build on the learnings of multiple successful prison-based HCV projects and initiatives that have emerged since direct-acting antivirals have become available (including in some LMICs where implementers lack resources to publish evaluation findings). By incorporating the needs, expertise, and knowledge of potential end-users in the design of the Toolkit, we have enhanced its potential for accessibility, relevance, and usability (Ramaswamy & Ozcan, 2014; Vargas et al., 2022). The result has been the development of well-informed resources to help advocates communicate their messages clearly and persuasively to relevant key audiences.

Our study has some limitations. As highlighted in our previous article (Walker et al., 2024), the findings cannot be generalised across all countries, given the relatively small sample size. Inclusion in the study required English proficiency which limited the participation of people from LMICs. We recognise that participant input did not extend to the co-design of research tools in Stage 1, however, we recommend future projects of this kind could benefit from involving community stakeholders in the design of recruitment methods, and the development of survey and interview instruments. We also acknowledge that social desirability bias was possible because surveys were distributed primarily through the networks and mailing lists of INHSU. Ongoing monitoring and evaluation of the Toolkit, however, once it has been launched and implemented, will help to address these limitations. That is, data will continue to be gathered to measure the success of the Toolkit - for reaching the breadth of audiences it aims to target, and meeting their needs on the continuum of change and in ways that match their in-country political, economic, and cultural contexts.

## Conclusion

All people in prison who are at risk of or living with HCV should have timely access to HCV testing and treatment services while incarcerated. As countries continue to strive for HCV elimination by 2030, the Toolkit should help guide and respond to the call for an increased focus on carceral settings. The Toolkit holds promise as a means of influencing and mobilising support and action for enhanced HCV testing and treatment services in prisons globally.

## Supplementary Material

Supplementary Material

Supplementary Material 2

Supplementary materials

Supplementary material associated with this article can be found, in the online version, at doi:10.1016/j.drugpo.2024.104628.

## Figures and Tables

**Table 1:** Summary of the co-design process that informed Toolkit development.

**Stage 1. Scoping study to determine advocacy needs**	Designed, distributed, and analysed online quantitative survey. Selected 10 participating countries. Designed qualitative interview guides. Recruited 25 key stakeholders for participation in qualitative interviews. Conducted qualitative interviews and thematically analysed data. Merged quantitative & qualitative data.
**Stage 2. Identifying target audiences and mapping resources using the**	Established three key audiences and key actions for the Framework.
**Advocacy Strategy Framework**	Examined interview data for suggested advocacy resources. Mapped suggested advocacy resources onto the Framework.
**Stage 3: Co-developing and validating resources**	Identified and developed a list of 7–12 tailored resources for each country. Emailed resource lists to participants (*n* = 25) for first round of review and feedback. Used feedback to finalise resource lists for each country. Sourced, adapted, and developed draft resources for each country. Emailed draft resources to participants for second round of review and feedback. Adapted resources based on participant feedback. Emailed near-final resources to participants for third/final review and feedback. Adapted resources based on final feedback as required. Final resources endorsed and incorporated into web-based Toolkit platform.

**Table 2:** Suggested advocacy resources for the Toolkit survey data.

Resource	LIC *n* = 55^1^	MIC *n* = 16^1^	HIC *n* = 110^1^	Total *n* = 181^1^	p-value^2^

Best practice case studies (videos)	34 (62 %)	10 (63 %)	61 (55 %)	105 (58 %)	0.7
Best practice case studies (written)	28 (51 %)	9 (56 %)	44 (40 %)	81 (45 %)	0.3
Infographics	23 (42 %)	11 (69 %)	55 (50 %)	89 (49 %)	0.2
Email letter templates	12 (22 %)	4 (25 %)	16 (15 %)	32 (18 %)	0.3
Fact sheets	27 (49 %)	9 (56 %)	62 (56 %)	98 (54 %)	0.7
Media releases or action alerts	29 (53 %)	7 (44 %)	40 (36 %)	66 (36 %)	0.13
Social media templates	28 (51 %)	5 (31 %)	27 (25 %)	60 (33 %)	0.003
Advocacy strategy guidelines	41 (75 %)	10 (63 %)	47 (43 %)	98 (54 %)	<0.001

1 *n* (%).

2 Fisher’s exact test, Pearson’s Chi-squared test.

* LIC = low-income country; MIC = middle-income country; HIC = high-income country.

**Table 3:** Suggested advocacy resources by target audience and changes needed.

Target audience	Changes needed	Suggested advocacy resources	Country income level*

**Policymakers & Funders**	Awareness	HCV prison prevalence & cost effectiveness modelling data	L M H
		Social media templates	L M
		Policy briefs about HCV in prisons	L M
	Will	Fact sheets about how to leverage funding	L M
		HCV prison strategy templates	L M
		Fact sheets about how to build advocacy campaigns	L M
	Action	Samples of supportive laws and policies	L M
		Monitoring & evaluation tools for HCV initiatives	L M
**Implementers**	Awareness	HCV infographics	L M H
		Videos of basic HCV information	L M
		Factsheets to address HCV stigma	L M H
	Will	Promotional networks for sharing knowledge & collaboration	L M
		Case studies of evidence-based models of HCV care	L M H
	Action	Resources about other blood borne viruses	L M
		Samples of up-to-date HCV clinical guidelines	L M
**Community**	Awareness	HCV infographics	L M H
		Fact sheets to address HCV stigma	L M H
		Downloadable fact sheets, booklets, and posters	L M H
	Will	Case studies & evaluations of peer-led HCV models of care	L M H
		Videos of peer stories of HCV testing & treatment	L M H
	Action	Tips on how to improve post-release transitional HCV care	L M H
		Case studies about capitalising on HIV services in the community	L M H

* *L* = low-income country; *M* = middle-income country; *H* = high-income country.

## Footnotes

2 World Bank Group income level country classifications are used for Jul 2023 - Jun 2024. For study purposes ‘LICs’ include low-income and lower middle-income countries; and ‘MICs’ include upper-middle income countries only. https://blogs.worldbank.org/opendata/new-world-bank-country-classifications-income-level-2022-2023

## References

[R1] Akiyama MJ, Kronfli N, Cabezas J, Sheehan Y, Scheibe A, Brahni T, & Luhmann N (2022). The role of low-income and middle-income country prisons in eliminating hepatitis C. The Lancet Public Health, 7(7), e578–e579. 10.1016/S2468-2667(22)00119-0 35779538 PMC9253889

[R2] Akiyama MJ, Kronfli N, Cabezas J, Sheehan Y, Thurairajah PH, Lines R, & Lloyd AR (2021). Hepatitis C elimination among people incarcerated in prisons: Challenges and recommendations for action within a health systems framework. The Lancet Gastroenterology & Hepatology, 6(5), 391–400. 10.1016/S2468-1253(20)30365-4 33857445 PMC8118192

[R3] Avery B, & Bashir S (2003). The road to advocacy—Searching for the rainbow. American Journal of Public Health, 93(8), 1207–1210. 10.2105/2Fajph.93.8.1207 12893596 PMC1447938

[R4] Caring Ambassadors Program. (2022). Hepatitis C advocacy toolkit. https://hepcchallenge.org/advocate/advocacy-tool-kit/.

[R5] Centre for Alcohol and Other Drug Training and Workforce Development. (2023). The Viral Hepatitis Toolkit. https://insight.qld.edu.au/toolkits/viral-hepatitis/detail.

[R6] Clarke V, & Braun V (2017). Thematic analysis. The Journal of Positive Psychology, 12 (3), 297–298.

[R7] Coffman J, & Beer T (2015). The advocacy strategy framework. Center for Evaluation Innovation. https://www.evaluationinnovation.org/wp-content/uploads/2015/03/Adocacy-Strategy-Framework.pdf.

[R8] Cohen BE, & Marshall SG (2017). Does public health advocacy seek to redress health inequities? A scoping review. Health & Social Care in the Community, 25(2), 309–328. 10.1111/hsc.12320 26749000

[R9] Degenhardt L, Webb P, Colledge-Frisby S, Ireland J, Wheeler A, Ottaviano S, & Hajarizadeh B (2023). Epidemiology of injecting drug use, prevalence of injecting-related harm, and exposure to behavioural and environmental risks among people who inject drugs: A systematic review. The Lancet Global Health, 11(5). 10.1016/s2214-109x(23)00057-8 PMC12580156 36996857

[R10] Dolan K, Wirtz AL, Moazen B, Ndeffo-Mbah M, Galvani A, Kinner SA, & Maher L (2016). Global burden of HIV, viral hepatitis, and tuberculosis in prisoners and detainees. The Lancet, 388(10049), 1089–1102. 10.1016/s0140-6736(16)30466-4 27427453

[R11] Gen S, & Wright AC (2013). Policy advocacy organizations: A framework ing theory and practice. Journal of Policy Practice, 12(3), 163–193.

[R12] Gill S, & Freedman T (2014). Climbing the Mountain: An approach to planning and evaluating public-policy advocacy. The Foundation Review, 6(3), 7. 10.9707/1944-5660.1211.

[R13] Glass J (2017). Advocates change the world; evaluation can help”: A literature review and key insights from the practice of advocacy evaluation. Canadian Journal of Program Evaluation, 32(1), 46–64.

[R14] Harm Reduction International (HRI). (2023). HRI Annual Report 2023. https://hri.global/wp-content/uploads/2024/04/HRI_AnnualReport_2023_Final-1.pdf.

[R15] HepVu. (2023). Hepatitis awareness month toolkit. Emory University. https://hepvu.org/hepatitis-awareness-month-toolkit-2023/.

[R16] International Network on Health and Hepatitis in Substance Users. (2024). Hepatitis C in primary care and drug and alcohol settings education program. https://www.inhsu.org/what-we-do/education/.

[R17] Klugman B (2011). Effective social justice advocacy: A theory-of-change framework for assessing progress. Reproductive Health Matters, 19(38), 146–162. 10.1016/s0968-8080(11)38582-5 22118149

[R18] Kronfli N, Buxton JA, Jennings L, Kouyoumdjian F, & Wong A (2019). Hepatitis C virus (HCV) care in Canadian correctional facilities: Where are we and where do we need to be? Canadian Liver Journal, 2(4), 171–183. 10.3138/2Fcanlivj.2019-0007 35992759 PMC9202815

[R19] Kronfli N, Dussault C, Bartlett S, Fuchs D, Kaita K, Harland K, & Cox J (2021). Disparities in hepatitis C care across Canadian provincial prisons: Implications for hepatitis C micro-elimination. Canadian Liver Journal, 4(3), 292–310. 10.3138/2Fcanlivj-2020-0035 35992251 PMC9202774

[R20] Laari L, & Duma SE (2023). Barriers to nurses health advocacy role. Nursing Ethics, 09697330221146241. 10.1177/09697330221146241. 36999769

[R21] Lafferty L, Rance J, Dore G, Grebely J, Lloyd A, Treloar C, & SToP-C Study Group. (2021). Hepatitis C treatment as prevention in the prison setting: Assessments of acceptability of treatment scale up efforts by prison correctional and health personnel. International Journal of Drug Policy, 98, Article 103379. 10.1016/j.drugpo.2021.103379 34311138

[R22] Lafferty L, Rance J, Grebely J, Lloyd AR, Dore GJ, Treloar C, & SToP-C Study Group. (2018). Understanding facilitators and barriers of direct-acting antiviral therapy for hepatitis C virus infection in prison. Journal of Viral Hepatitis, 25(12), 1526–1532. 10.1111/jvh.12987 30141261

[R23] Loue S (2006). Community health advocacy. Journal of Epidemiology & Community Health, 60(6), 458–463. 10.1136/jech.2004.023044 16698972 PMC2563937

[R24] Nakitanda AO, Montanari L, Tavoschi L, Mozalevskis A, & Duffell E (2020). Hepatitis C virus infection in EU/EEA and United Kingdom prisons: Opportunities and challenges for action. BMC Public Health, 20(1), 1–12. 10.1186/s12889-020-09515-6 33167912 PMC7650151

[R25] National Alliance of State and Territorial AIDS Directors. (2011). Viral hepatitis advocacy toolkit. https://www.globalhep.org/sites/default/files/content/resource/files/2020-01/NASTAD%20Viral%20Hepatitis%20Advocacy%20Kit.pdf.

[R26] Ocal S, Muir AJ, & Naggie S (2019). Hepatitis C behind and beyond bars: Targeting the US prison population and changing North Carolina prisoner health policy. North Carolina Medical Journal, 80(6), 352–355. 10.18043/ncm.80.6.352 31685570

[R27] Papaluca T, Hellard ME, Thompson AJ, & Lloyd AR (2019). Scale-up of hepatitis C treatment in prisons is key to national elimination. Medical Journal of Australia, 210(9), 391–393. 10.5694/mja2.50140. e391. 30968417

[R28] Pawlotsky J-M, Negro F, Aghemo A, Berenguer M, Dalgard O, & Dusheiko G (2020). EASL recommendations on treatment of hepatitis C: Final update of the series. Journal of Hepatology, 73(5), 1170–1218. 10.1016/j.jhep.2020.08.018 32956768

[R29] Ramaswamy V, & Ozcan K (2014). The co-creation paradigm. Stanford University Press.

[R30] Rockstroh JK, Swan T, Chang J, Elamouri F, & Lloyd AR (2023). The path to hepatitis C elimination: Who are we leaving behind and why? Journal of the International AIDS Society, 26(7). 10.1002/jia2.26136 PMC10371387 37494827

[R31] Sánchez de la Guía L, Puyuelo Cazorla M, & de-Miguel-Molina B (2017). Terms and meanings of “participation” in product design: From “user involvement” to “co-design. The Design Journal, 20(sup1), S4539–S4551. 10.1080/14606925.2017.1352951

[R32] Slattery P, Saeri AK, & Bragge P (2020). Research co-design in health: A rapid overview of reviews. Health Research Policy and Systems, 18, 1–13. 32046728 10.1186/s12961-020-0528-9 PMC7014755

[R33] Stone J, Fraser H, Lim AG, Walker JG, Ward Z, MacGregor L, & Abramovitz D (2018). Incarceration history and risk of HIV and hepatitis C virus acquisition among people who inject drugs: A systematic review and meta-analysis. The Lancet Infectious Diseases, 18(12), 1397–1409. 10.1016/S1473-3099(18)30469-9 30385157 PMC6280039

[R34] Stöver H, Meroueh F, Marco A, Keppler K, Saiz De La Hoya P, Littlewoood R, & Somaini L (2019). Offering HCV treatment to prisoners is an important opportunity: Key principles based on policy and practice assessment in Europe. BMC Public Health, 19(1), 1–11. 10.1186/s12889-018-6357-x 30621658 PMC6323720

[R35] Survey Monkey Inc. (2023). https://www.surveymonkey.com.

[R36] Vargas C, Whelan J, Brimblecombe J, & Allender S (2022). Co-creation, co-design, co-production for public health: A perspective on definition and distinctions. Public Health Research & Practice, 32(2). 10.17061/phrp3222211 35702744

[R37] Walker SJ, Shrestha LB, Lloyd AR, Dawson O, Sheehan Y, & Kronfli N (2024). Barriers and advocacy needs for hepatitis C services in prisons: Informing the prisons hepatitis C advocacy toolkit. International Journal of Drug Policy, 126, Article 104386. 10.1016/j.drugpo.2024.104386 38492433 PMC11106844

[R38] Winter R, Holmes J, Papaluca T, & Thompson A (2022). The Importance of prisons in achieving hepatitis C elimination: Insights from the Australian experience. Viruses, 14(497), 1–15. 10.3390/2Fv14030497 PMC8949789 35336905

[R39] World Health Organization. (2016). Global health sector strategy on viral hepatitis 2016–2021: Towards ending viral hepatitis. https://www.who.int/publications/i/item/WHO-HIV-2016.06.

[R40] World Health Organization. (2019). Access to hepatitis C testing and treatment for people who inject drugs and people in prisons — A global perspective. https://www.who.int/publications/i/item/WHO-CDS-HIV-19.6.

[R41] World Health Organization. (2024). Global hepatitis report 2024: Action for access in low- and middle-income countries. Geneva: World Health Organization. https://www.who.int/publications/i/item/9789240091672.

[R42] World Hepatitis Alliance. (2018). NOhep Advocacy Toolkit. Race to 2030: Accelerating action at a national level. https://www.nohep.org/wp-content/uploads/2020/01/Race-to-2030-Advocacy-Toolkit-online-FINAL.pdf.

