# Supplementary material for: Co-designing the INHSU Prisons Hepatitis C Advocacy Toolkit using the Advocacy Strategy Framework
Source: Int J Drug Policy. Author manuscript; Available in PMC 2026 Jan 31. (PMC12860404; doi:10.1016/j.drugpo.2024.104628)
Supplement: Supplementary Material [file NIHMS2132262-supplement-Supplementary_Material.docx]

**SUPPLEMENTARY MATERIAL**

**Appendix S1: Scoping study interview schedule**

| Participant ID and Code |  |
| --- | --- |
| Date and time of interview |  |
| Duration of interview |  |
| Country location / income level |  |
| Participant role and gender |  |

- Introductions. Thank participant for completing survey.
- Explain what is meant by “Advocacy”: actions or steps to address an issue or problem to bring about positive change.
- Explain the aim of the Toolkit: Selection of advocacy tools and resources to enhance hepatitis C services (testing/treatment) in prisons globally, that will be housed on a website for easy access.
- Explain aim of interviews - to gain in-depth insights and views from people like you, to make sure advocacy Toolkit resources we develop are as useful as possible, for the purpose of advocating for increased and improved hep C programs and services in prisons.
- Reminder about confidentiality (audio files, transcripts, de-identifying data)
- Any questions?

| **Participant role** | Can you tell me about your role?   - What do you do in your role? - How does it relate to hepatitis C programs in prisons in your country? |
| --- | --- |
| - Notes from participant survey exported here to refer to during interview | |
| **Country context re hep C programs in prisons** | What’s available in terms of prison-based hep C programs in your country?   - Who employs/funds the healthcare workforce? Govt, NGOs, both? - How are programs implemented/who oversees their implementation? - Who provides healthcare in prisons? Physicians, nurses, both? - Available in all prisons? (why/why not?) - Who funds and leads these programs? - Other health programs (e.g., HIV, drug treatment) |
| - Notes from participant survey exported here to refer to during interview | |
| **Gaps in hep C programs** | What do you think are the 3 biggest service gaps regarding hepatitis C services in prisons? |
| - Notes from participant survey exported here to refer to during interview | |
| **Advocacy resources needed to address gaps** | Thinking about these service gaps you’ve just described, WHAT tools or resources do you think could be helpful for advocating for increasing people in prisons access to screening and treatment in Scotland? |
| - Notes from participant survey exported here to refer to during interview | |
| Are there any other things you’d like to share – in relation to the Prisons Advocacy Toolkit?  Thank you for sharing your experience and views. | |
