# Supplementary figures and images for: Co-designing the INHSU Prisons Hepatitis C Advocacy Toolkit using the Advocacy Strategy Framework
Source: Int J Drug Policy. Author manuscript; Available in PMC 2026 Jan 31. (PMC12860404; doi:10.1016/j.drugpo.2024.104628)

SUPPLEMENTARY MATERIAL

Appendix S2: Advocacy Strategy Framework

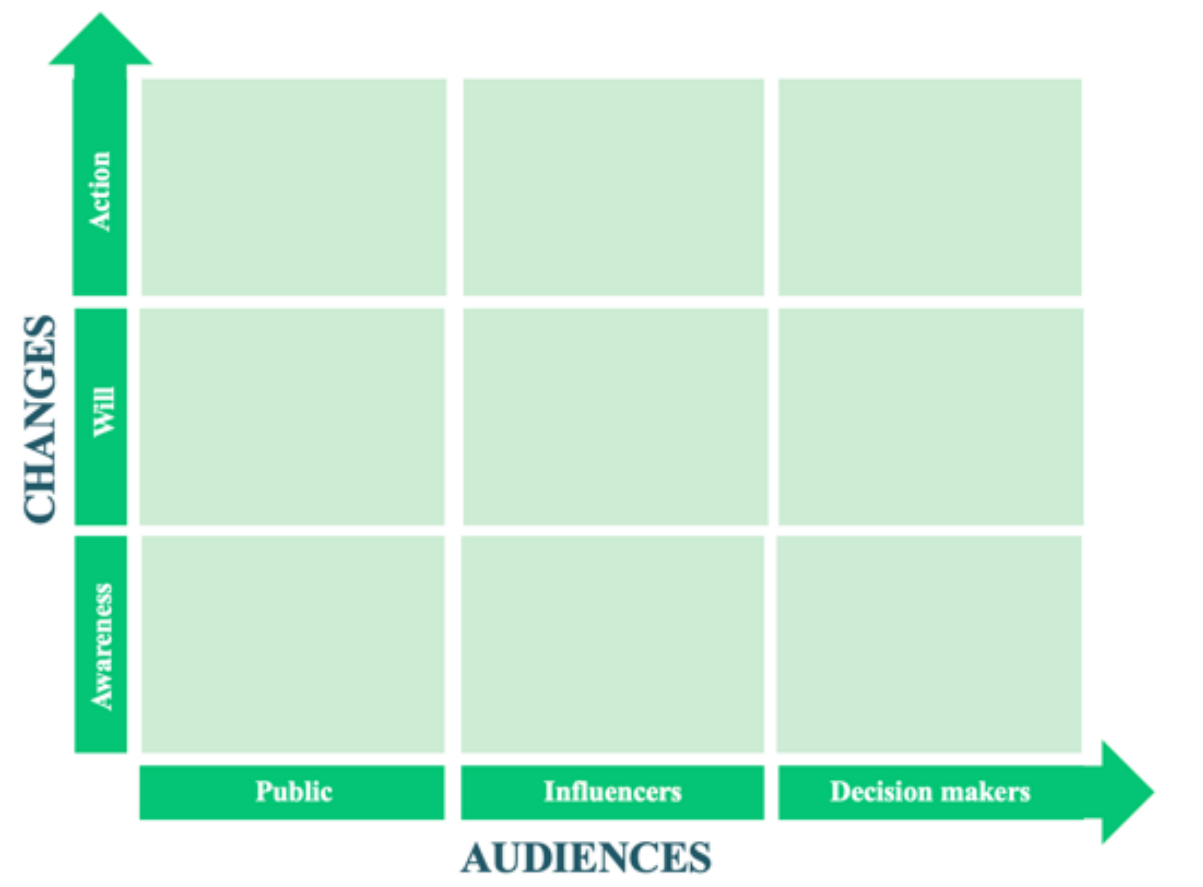

Supplement: Supplementary Material 2 [file NIHMS2132262-supplement-Supplementary_Material_2.pdf]
